# Tyrosine phosphatase SHP2 exacerbates psoriasis‐like skin inflammation in mice via ERK5‐dependent NETosis

**DOI:** 10.1002/mco2.120

**Published:** 2022-03-04

**Authors:** Yan Ding, Zijun Ouyang, Chenyang Zhang, Yuyu Zhu, Qiang Xu, Haiyan Sun, Jiao Qu, Yang Sun

**Affiliations:** ^1^ State Key Laboratory of Pharmaceutical Biotechnology, Department of Biotechnology and Pharmaceutical Sciences, School of Life Sciences Nanjing University Nanjing China; ^2^ Institute of Marine Biomedicine, School of Food and Drug Shenzhen Polytechnic Shenzhen Guangdong China; ^3^ Chemistry and Biomedicine Innovation Center (ChemBIC) Nanjing University Nanjing China

**Keywords:** ERK5, NETosis, NETs, PTPN11, single‐cell RNA sequencing

## Abstract

Psoriasis is a chronic inflammatory skin disease, often accompanied by increased infiltration of immune cells, especially neutrophils. However, the detailed mechanism of the neutrophil function in psoriasis progression remains unclear. Here, we found that both Src homology‐2 domain‐containing protein tyrosine phosphatase‐2 (SHP2) and neutrophils were highly correlated to developing psoriasis by single‐cell ribonucleic acid (RNA) sequencing and experiment verification. The deficiency of SHP2 in neutrophils significantly alleviated psoriasis‐like phenotype in an imiquimod‐induced murine model. Interestingly, high levels of neutrophil extracellular traps (NETs) were produced in the inflamed lesions of psoriatic patients. In addition, imiquimod‐induced psoriasis‐like symptoms were remarkably ameliorated in peptidyl arginine deiminase 4 (PAD4) knockout mice, which cannot form NETs. Mechanistically, RNA‐seq analysis revealed that SHP2 promoted the formation of NETs in neutrophils via the ERK5 pathway. Functionally, this mechanism resulted in the infiltration of pro‐inflammatory cytokines such as TNF‐*α*, IL‐1*β*, IL‐6, IL‐17A, and CXCL‐15, which enhances the inflammatory response in skin lesions and reinforces the cross‐talk between neutrophils and keratinocytes, ultimately aggravating psoriasis. Our findings uncover a role for SHP2 in NET release and subsequent cell death known as NETosis in the progression of psoriasis and suggest that SHP2 may be a promising therapeutic target for psoriasis.

## Graphical abstract

Single‐cell RNA sequencing and experimental verification were combined to announce that SHP2 aggravates psoriasis‐like skin inflammation in mice via ERK5‐dependent NETosis. During this process, inflammatory cytokines: TNF‐*α*, IL‐1*β*, IL‐6, IL‐17a, and CXCL‐15 were infiltrated in skin and contributed to psoriasis. Our study provides evidence for the role of SHP2 in NETosis and psoriasis. SHP2 may be a potential therapeutic target for the treatment of psoriasis.

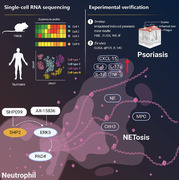

## 1 INTRODUCTION

Psoriasis is a common chronic relapsing inflammatory skin disease that is currently defined as autoimmune. Psoriatic lesions are clinically characterized by red plaques covered with silvery‐white scale and circumscribed papules, and patients usually present with itching and swelling.^1^ The histopathological features of psoriasis are hyperkeratosis of keratinocytes in the epidermal base, dilated and elongated capillaries in the dermal layer, and infiltration of inflammatory cells in the skin lesion area.^2^ Methotrexate and cyclosporine are two often prescribed medications for psoriasis. However, due to the risk of relapse upon treatment withdrawal and the potential for hazardous side effects, long‐term use of these drugs is restricted, resulting in the inability to completely cure psoriasis.^3^ In 2004, the era of biological psoriasis treatment began. Etanercept, the first TNF‐*α* inhibitor to treat psoriasis, was approved by the U.S. Food and Drug Administration. It is widely used clinically with its good therapeutic effect and relatively minor side effects.^4^ After that, researchers developed a variety of biological agents which targeted inflammatory factors in the pathogenesis of psoriasis, such as ixekizumab (IL‐17 inhibitors) and guselkumab (IL‐23 inhibitors) have been invented, they were reported to play a core role in targeting the IL‐23/Th17 axis.^5^ However, because costly biologics impose a significant economic burden on patients, we focused on low‐cost small molecule medicines and evaluated their potential for treating psoriasis. The dysfunctional immune system has been demonstrated to be a feature of psoriasis skin lesions and serves as the primary entrance point for study into the pathogenesis of psoriasis.^6^, ^7^, ^8^ As the most abundant cells in innate immune cells, neutrophils also play an essential role in the occurrence and development of psoriasis.^9^, ^10^

The traditional defense mechanisms of neutrophils include pathogen phagocytosis, degranulation, and cytokine production. Studies have recently found that neutrophils can undergo a unique cell death process called “NETosis” when stimulated by inflammatory factors, chemicals, or metabolites.^11^ In this process, peptidyl arginine deiminase 4 (PAD4) is responsible for the citrullination of histones in neutrophils, and histone 3 (H3) in the nucleus is modified to form citrullinated histone 3 (Cit‐H3), which in turn causes the chromatin to become loose.^12^ Subsequently, myeloperoxidase (MPO) and neutrophil elastase (NE), which enter the nucleus to cleave histones to separate them from DNA, cause further chromatin decondensation.^13^ At last, DNA is released to the outside of the cell.^14^ At the same time, the granular proteins in the cytoplasm, including enzymes and antibacterial peptides, attach to the scaffold composed of chromatin to form a network‐like complex called neutrophil extracellular traps (NETs). Studies have shown that NETs play a vital role in the pathogenesis of psoriasis.^15^

Src homology phosphotyrosine phosphatase 2 (SHP2) is a non‐transmembrane protein tyrosine phosphatase encoded by the human *PTPN11* gene and consists of two *N*‐terminal SH2 domains, a C‐terminal tail, and a catalytic protein tyrosine phosphatase (PTP) domain.^16^ SHP2 is involved in cell proliferation, differentiation, and survival. SHP2 is also implicated in the regulation of immune cells and the onset of inflammation, as evidence accumulates.^17^ For example, SHP2 positively regulates the oxidative burst of macrophages^18^ and promotes the migration of dendritic cells to secondary lymphoid organs.^19^ SHP2 can also bind to multiple receptors related to the immune response to activating T cells and participate in the downstream signal transduction of PD‐1.^20^ Noonan syndrome is a congenital heart disease mainly caused by gain of function mutations of SHP2, and one patient was shown to have pustular psoriasis, suggesting a functional correlation between phosphatase activity and psoriasis.^21^

The mitogen‐activated protein kinase (MAPK) signaling pathway is one of the critical signal transduction systems in organisms. Its subfamilies mainly include extracellular signal regulated kinase 1/2 (ERK1/2), c‐jun amino terminal kinase (JNK), p38 mitogen activated protein kinase (p38 MAPK), and extracellular signal regulated kinase (ERK5).^22^ The MAPK family can participate in cell proliferation, differentiation, apoptosis, and other functions and has been associated with NETs formation.^23^ ERK5 is encoded by *MAPK7* in humans, and expressed in most human cells, and can be activated by mechanical stimulation, oxidative stress, hypertonic environment, cytokines, and inflammatory cytokines (such as IL‐6).^24^ ERK5 widely participates in and regulates human diseases, including Alzheimer's disease,^25^ breast cancer,^26^ and atherosclerosis.^27^

It has been established that decreasing SHP2 can improve psoriasis symptoms,^28^ and NETosis can accelerate the process of psoriasis.^29^ However, the mechanism by which SHP2 modulates neutrophil NETosis remains unclear. Here, we use the imiquimod (IMQ)‐induced psoriasis mouse model to investigate the relationship between SHP2 and NETosis in neutrophils. In addition to that, RNA‐seq and single‐cell RNA sequencing technology were also used for better experimental guidance. Based on these data, we propose that SHP2 in neutrophils promotes the release of NETs through the ERK5 pathway, which aggravates psoriasis.

## 2 RESULTS

### 2.1 Single‐cell RNA sequencing revealed that neutrophils and SHP2 were related to developing psoriasis

**FIGURE 1:**
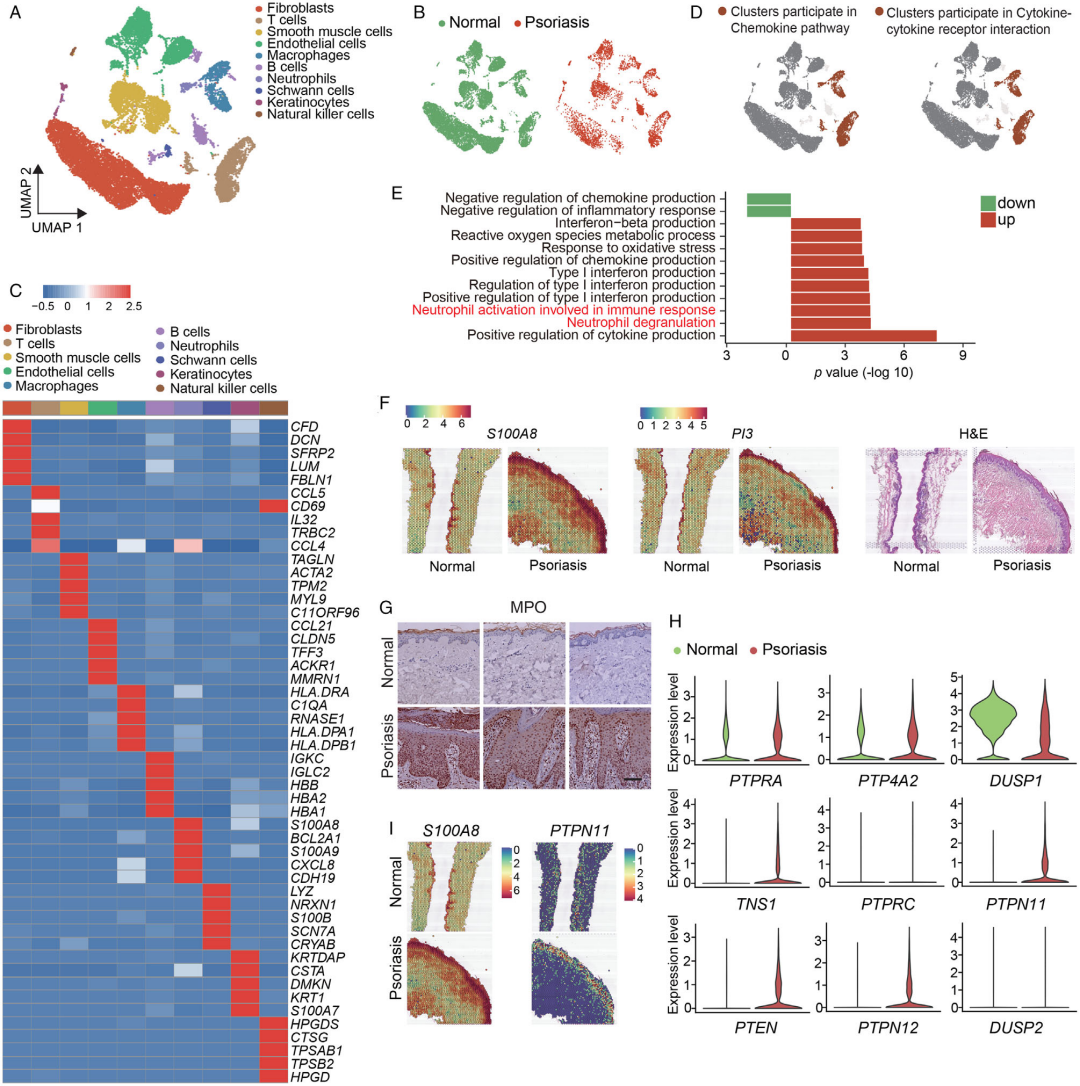
Single‐cell RNA sequencing reveals that SHP2 expression is related to developing psoriasis. (A) Unbiased clustering of human skin data shown by UMAP plot. (B) UMAP visualization of the distribution of cells splitting by samples. (C) Heatmap demonstrated the top five differentially expressed genes for each cluster. (D) Gene set enrichment analysis (GSEA) of different clusters and shown by UMAP plot. (E) *GO* pathways analysis through top genes from human skin tissue. (F) The hematoxylin and eosin (H&E) staining of the skin sections and spatial feature plot of *PI3* and *S100A8*’s expression. (G) Representative immunohistochemical image staining for myeloperoxidase (MPO) of healthy (*n* = 8) and psoriasis patients (*n* = 13) donors, scale bar = 400 μm. (H) The violin plot of the top nine expression abundance of 107 protein tyrosine phosphatases (PTPs). (I) The spatial feature plot of *S100A8* and *PTPN11*’s expression

We first collected skin tissue from healthy donors and psoriasis patients, isolated them into single cells, and performed single‐cell RNA sequencing to explore the contributions of various cell groups to psoriasis. Figure 1A showed the cell groups in human skin, including fibroblasts, smooth muscle cells, endothelial cells, Schwann cells, and keratinocytes, among which immune‐related cells included T cells, macrophages, B cells, neutrophils, and natural killer cells. In Figure 1B, we showed the distribution of cells of skin tissues from normal people and psoriasis patients. The heat maps of functional genes of various cell groups are shown in Figure 1C. Then, the chemokine and cytokine signaling pathway were explored and found out that neutrophils also contributed to them in addition to T cells and macrophages (Figure 1D). Pathways enriched by top genes in psoriasis patients and normal people with GO analysis were analyzed. As we saw in Figure 1E, compared to normal people, pathways related to inflammation, neutrophil activation, and degranulation were up‐regulated. While, pathway related to inflammation's negative regulation was down‐regulated. This result suggested that the skin tissue of psoriasis patients was in an inflammation state, which infiltered with cytokines, interferons, chemokine, reactive oxygen species, and neutrophils. Then, we conducted spatial transcriptomics analysis using normal and psoriasis skin tissue. According to the expression of neutrophil marker genes, skin tissue domain was colored and showed that *PI3* and *S100A8*, two neutrophil marker genes, are highly expressed in psoriasis patients (Figure 1F). Next, we explored the distribution of MPO proteins in skin tissue. Immunohistochemical staining of the skin sections from patients with psoriasis and healthy donors was performed. Results showed that the infiltration of neutrophils in psoriasis lesions was significantly higher than that in healthy donors (Figure 1G). In conclusion, our data can infer that neutrophils are highly expressed in the skin tissue of patients with psoriasis, which may contribute to the pathogenesis of psoriasis.

Next, the expression levels of 107 genes that encode human PTP phosphatase family members were displayed (Figure S1) and showed the top nine in Figure 1H. Results showed that the SHP2 coding gene *PTPN11* expressed more in the skin from psoriasis patients than from healthy donors. The results in Figure 1I showed that the expression of *S100A8 and PTPN11* is increased significantly in patients’ skin tissue. In conclusion, these evidences suggest that neutrophils and SHP2 may play a key role in psoriasis.

### 2.2 The deficiency of SHP2 improves psoriasis‐like phenotype in the mice model

**FIGURE 2:**
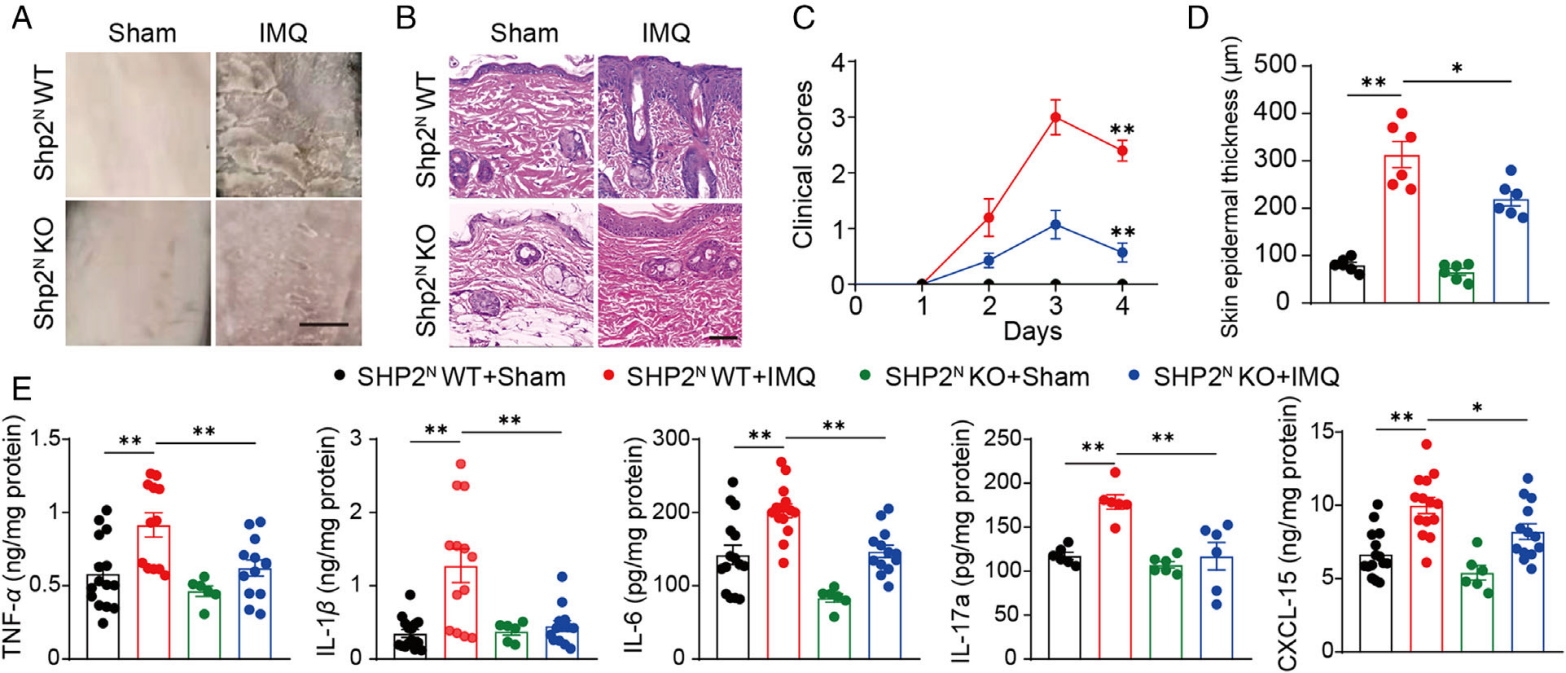
Src homology‐2 domain‐containing protein tyrosine phosphatase (SHP2) deficiency in neutrophils alleviates psoriasis‐like phenotype in the imiquimod (IMQ)‐induced murine model. (A) Phenotypic presentation of back skin sections of sham‐ or IMQ‐treated SHP2^N^ KO and WT mice, scale bar = 5 mm. (B) H&E staining of back skin sections of sham‐ or IMQ‐treated SHP2^N^ KO and WT mice, scale bar = 200 μm. Clinical scores (C) and epidermal thickness (D) of mice dorsal skin. (E) Dorsal skin was infiltrated with TNF‐*α*, IL‐1*β*, IL‐6, IL‐17a, and CXCL‐15 evaluated by ELISA

To explore the function of SHP2 on psoriasis, SHP099 (10 mg/kg) was injected into mice, which is an effective SHP2 inhibitor. Results showed that the symptoms of psoriasis induced by imiquimod (IMQ) were improved by SHP2 inhibition (Figure S2). Since neutrophils have increased in patients with psoriasis, and SHP2 inhibition can alleviate psoriasis, we crossed *Shp2* ^flox/flox^ mice with *S100a8*cre mice and constructed Cre‐driven SHP2 deletion in neutrophils: *S100a8* ^cre^‐*Shp2* ^flox/flox^ (SHP2^N^ KO) mice and *Shp2* ^flox/flox^ (SHP2^N^ WT) mice to further study the role of SHP2 in psoriasis. The results showed that psoriasis symptoms caused by IMQ on the back of SHP2^N^ WT mice, such as scaly coverage and thickening of the epidermis, were significantly improved in SHP2^N^ KO mice (Figure 2A,B). The adapted human clinical Psoriasis Area and Severity Index (PASI) score of the IMQ‐treated SHP2^N^ KO mice was significantly lower than IMQ‐treated SHP2^N^ WT mice (Figure 2C). Accordingly, the increase of epidermal thickness caused by IMQ was also alleviated in SHP2^N^ KO mice (Figure 2D). In addition, the expression of inflammatory factors TNF‐*α*, IL‐1*β*, IL‐6, IL‐17a, and CXCL‐15 was significantly reduced in SHP2^N^ KO mice's skin (Figure 2E), which meant that the inflammatory environment in mice skin tissue has been improved. In summary, our data indicate that the inhibition of SHP2 or specific deletion of SHP2 in neutrophils can alleviate IMQ‐induced psoriasis symptoms.

### 2.3 NETs can mediate the pathogenesis of psoriasis are associated with SHP2

NETosis is one of the main mechanisms of neutrophil defense and plays a crucial role in inflammatory diseases. We used skin sections from normal people and psoriasis patients to conduct spatial transcriptomics analysis. As shown in Figure S3A, there are 14 cell groups, and their marker genes are displayed in the dot plot in Figure S3C. *CASP4* and *PPIF*, two marker genes that relate to the formation of NETs, were found out to be expressed more in psoriatic skin tissue (Figure S3B).

**FIGURE 3:**
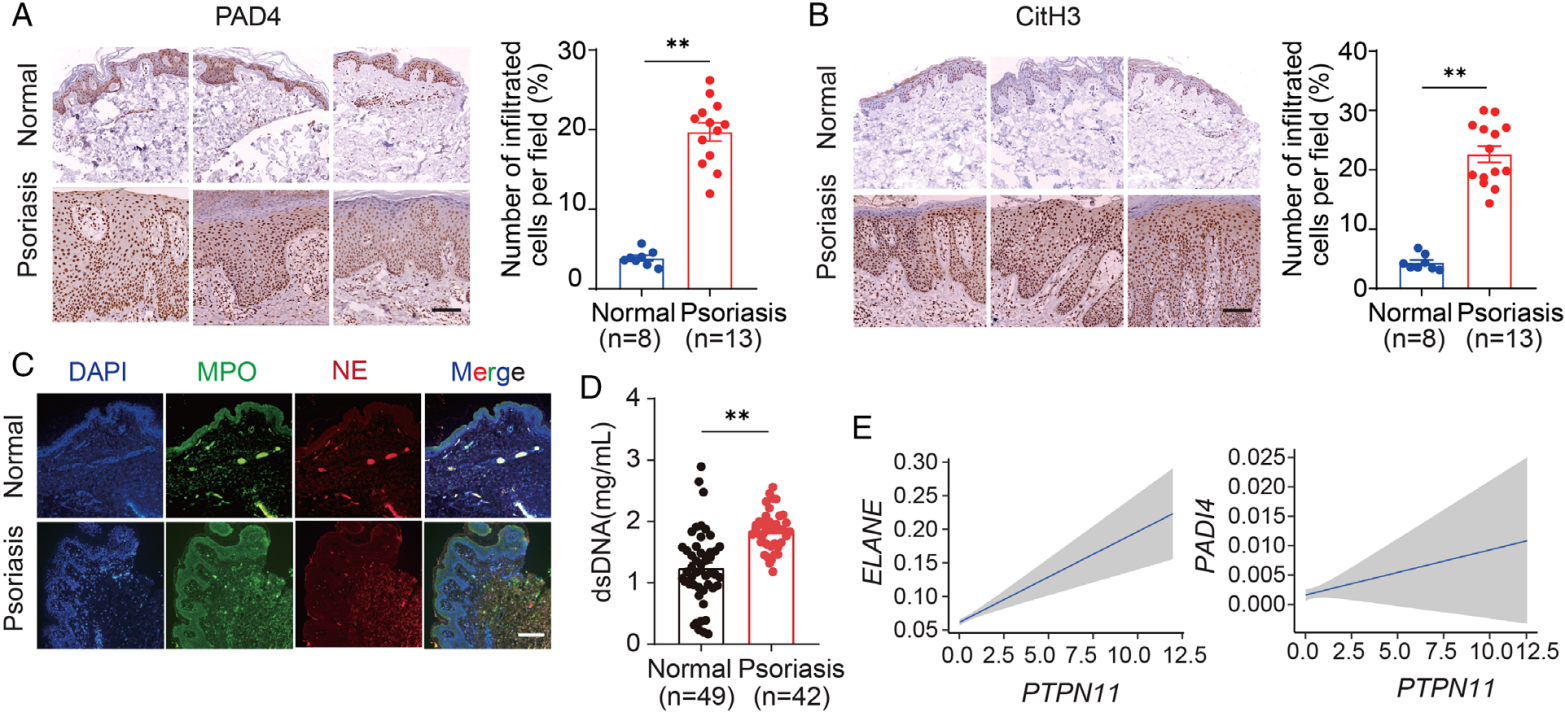
Neutrophil extracellular traps (NETs) can mediate the pathogenesis of psoriasis associated with SHP2. Representative immunohistochemical staining of skin sections from psoriasis patients and healthy donors with anti‐PAD4 (A) or anti‐CitH3 (B), scale bar = 400 μm. (C) Representative immunofluorescence staining of skin sections from healthy donors (*n* = 8) and psoriasis patients (*n* = 13) with anti‐MPO, anti‐neutrophil elastase (NE), and 4′,6‐diamidino‐2‐phenylindole (DAPI), scale bar = 400 μm. (D) Quantification of dsDNA in serum samples from healthy and psoriasis donors. (E) The expression level of *PTPN11, ELANE*, and *PADI4* in human skin

To explore how NETs contributed to psoriasis, we collected the skin tissues of psoriasis patients and healthy donors and detected PAD4 (Figure 3A) and CitH3 (Figure 3B) protein expression, which are two critical proteins formed in the process of NETosis by immunohistochemistry. The results showed that the expression of CitH3 and PAD4 in skin lesions of psoriasis patients increased significantly compared with the skin of healthy donors. In addition, the immunofluorescence method was used to obtain images of NE and MPO in skin sections to observe NETs. The results showed that more NETs were produced in the skin of patients with psoriasis (Figure 3C). DsDNA, which could be indicative of the production of NETs, was quantified in serum from healthy individuals and psoriasis patients. The results indicated that dsDNA levels in psoriasis serum samples are significantly higher than in the normal people (Figure 3D). Finally, we analyzed genes’ mRNA expression of *ELANE*, *PADI4*, and *PTPN11* in human skin tissue. Data from single‐cell RNA sequencing showed that mRNA expression of *ELANE* and *PADI4* increased with the increase of *PTPN11* expression (Figure 3E). Taken together, these findings indicate that SHP2 and neutrophils are highly correlated to developing psoriasis.

### 2.4 Deletion of NETs can alleviate IMQ‐induced psoriasis symptoms in mice

**FIGURE 4:**
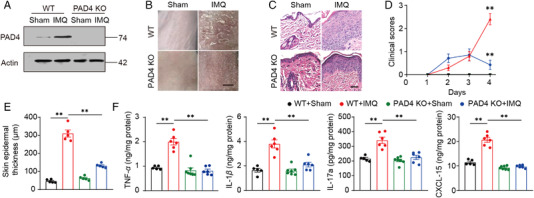
Deletion of neutrophil extracellular traps (NETs) can alleviate IMQ induced psoriasis symptoms in mice. (A) The protein level of PAD4 in mice dorsal skin, tested by western blot. (B) Phenotypic presentation of sham‐ or IMQ‐treated PAD4 KO and WT mice, scale bar = 5 mm. (C) H&E staining of back skin sections of sham‐ or IMQ‐treated PAD4 KO and WT mice, scale bar = 200 μm. Clinical scores (D) or epidermal thickness (E) of mice dorsal skin. (F) Dorsal skin was infiltrated with TNF‐*α*, IL‐1*β*, IL‐17a, and CXCL15 evaluated by ELISA

Next, *Padi4* knockout (PAD4 KO) mice were constructed which could not form NETs. To confirm the efficiency of PAD4 deletion, we dissociated the skin of PAD4 KO mice and examined the expression of PAD4 using western blot (Figure 4A). In the following experiment, PAD4 KO or WT mice were randomly divided into two groups treated with equal amounts of IMQ or control cream for four days. Skin tissue was obtained on day 4. Compared with WT mice, the deletion of *Padi4* significantly reversed the psoriasis‐like symptoms caused by IMQ. Scaling was significantly improved in PAD4 KO mice (Figure 4B). In addition, the epidermal hyperplasia was also significantly reduced, proven by epidermal thickness and hematoxylin‐eosin (H&E) staining, as we can see in Figure 4C,E. What's more, the PASI score of IMQ treated WT mice continued to increase and peaked on day 4, while the PASI score of PAD4 KO mice continually kept on a low value and decreased on day 4 (Figure 4D).

Furthermore, the expression of psoriasis‐related inflammatory cytokines was measured in mice's skin. The results showed that *Padi4* gene knockout effectively reversed the over‐secretion of inflammatory factors TNF‐*α*, IL‐1*β*, CXCL‐15, and IL‐17a on the skin of mice caused by IMQ (Figure 4F). These data suggest that NETs deficiency can alleviate IMQ‐induced psoriasis symptoms in mice.

### 2.5 Single‐cell RNA sequencing reveals that the inhibition of SHP2 can decrease neutrophil infiltration

**FIGURE 5:**
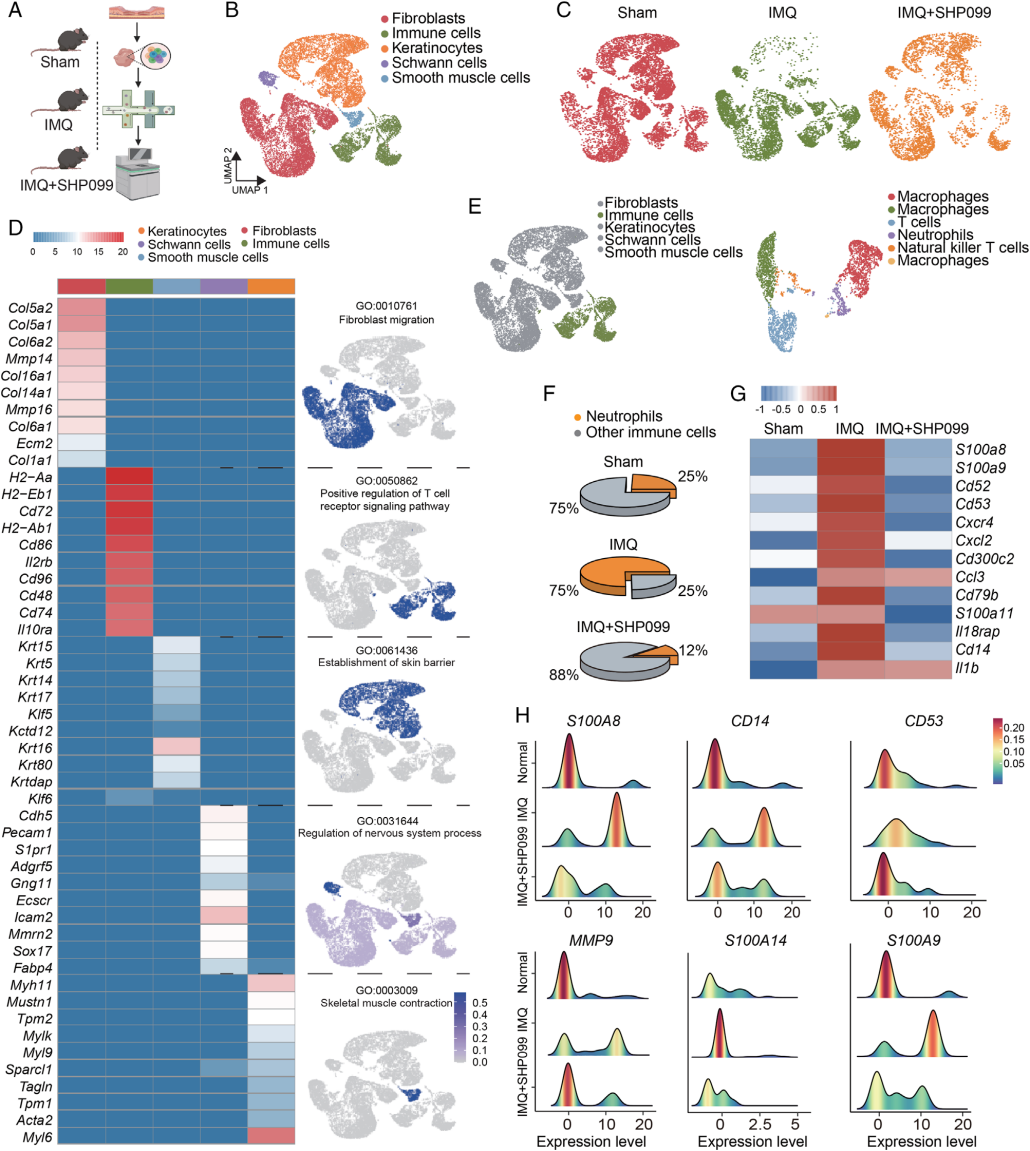
Single‐cell RNA sequencing reveals that the inhibition of SHP2 can decrease neutrophil infiltration. (A) Schematic representation of the experimental procedure. (B) Unbiased clustering reveals five cellular clusters. (C) Cell atlas of mice skin tissue. (D) Heat map image of the functional gene set and cell atlas of each cell population. (E) Unbiased clustering of immune cells. (F) Pie chart of the proportion of neutrophils to immune cells. (G) Heat map image of neutrophil‐related functional gene sets. (H) Summit plot of neutrophil‐related functional genes

We collected the skin tissues of mice treated with IMQ or SHP099 or none, dissociated them into single cells, and performed the single‐cell RNA sequencing (Figure 5A). Unbiased clustering revealed five cell clusters (Figure 5B), and the cell distribution was shown in Figure 5C. According to specific marker genes, there are fibroblasts, immune cells, keratinocytes, Schwann cells, and smooth muscle cells were defined (Figure 5D). Immunized cell subsets were defined as macrophages, T cells, neutrophils, and natural killer T cells (Figure 5E). Results showed that the proportion of neutrophils in immune cells increased significantly after IMQ treatment and decreased after SHP099 treatment (Figure 5F). Next, we analyzed neutrophil‐related genes. Results showed that neutrophil infiltration occurred in the skin tissues after IMQ treatment, and SHP099 significantly reversed this phenomenon (Figure 5G,H).

### 2.6 Inhibition or deletion of SHP2 can inhibit the formation of NETs

**FIGURE 6:**
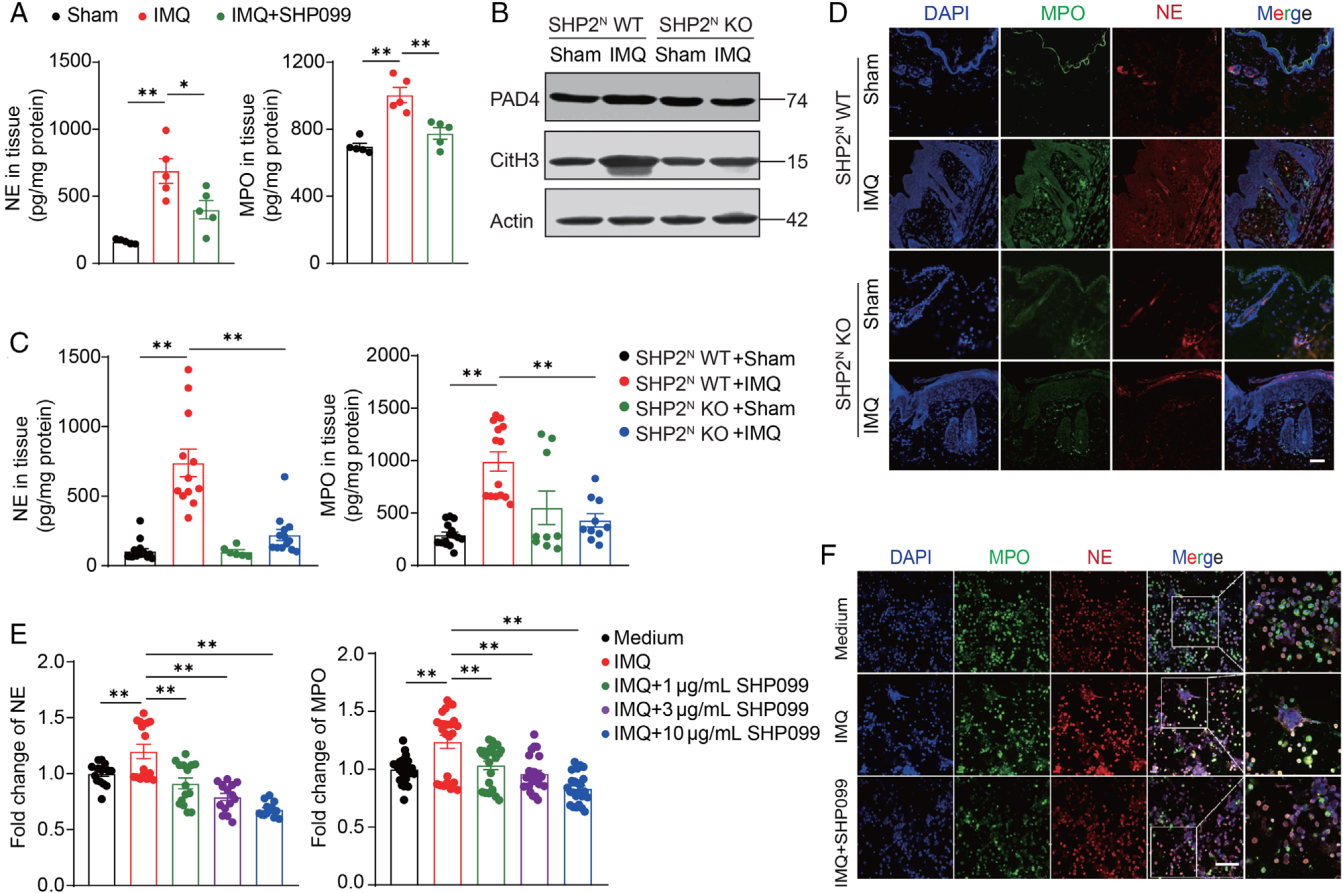
Inhibition of SHP2 expression in neutrophils can inhibit the formation of neutrophil extracellular traps (NETs). (A) The protein expression of NE and MPO in the IMQ and/or SHP099 treated mice. (B) The protein level of CitH3 and PAD4 of mice dorsal skin, tested by western blot. (C) The protein expression of NE and MPO of mice dorsal skin tested by ELISA. (D) Representative immunofluorescence images staining for MPO (green), NE (red), and DAPI (blue) of mice skin section, scale bar = 200 μm. (E) The protein expression of NE and MPO in neutrophils extracted from peripheral blood. (F) Representative immunofluorescence images staining for MPO (green), NE (red), and DAPI (blue) of neutrophils extracted from peripheral blood, scale bar = 100 μm

To determine the influence of SHP2 on NET formation, we first examined NE and MPO expression in mice treated with IMQ and/or SHP099. SHP2 inhibition resulted in a decrease in the expression of proteins NE and MPO (Figure 6A). Then, neutrophils specific SHP2 knockout mice were used to make some future exploration. The results showed that IMQ significantly increased the expression of PAD4 and CitH3, and *Shp2* specific knockout in neutrophils had revered this situation (Figure 6B). The same trend was proved in the expression of NE and MPO proteins detected by ELISA and immunofluorescence staining (Figure 6C,D). Next, we extracted neutrophils from the peripheral blood of healthy donors and then processed them with IMQ and/or SHP099. The results showed that IMQ treatment significantly increased the expression of NE and MPO, while SHP099 effectively inhibited the expression of these two proteins in a dose‐dependent manner (Figure 6E,F). These results preliminarily confirm that NETs involved in the progression of psoriasis can be down‐regulated by SHP2 inhibition or deletion.

### 2.7 SHP2 promotes the formation of NETs through the ERK5 pathway

**FIGURE 7:**
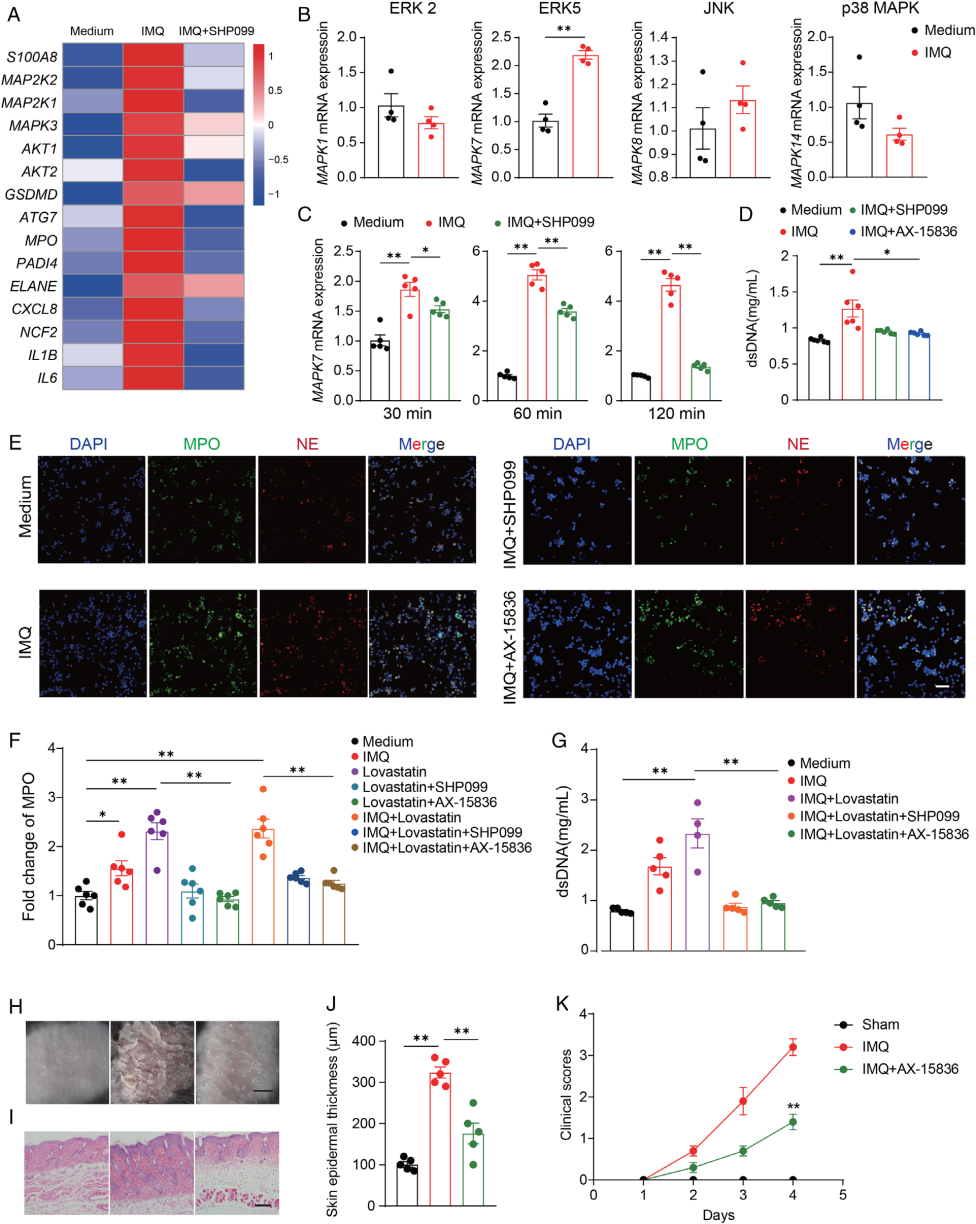
SHP2 promotes the formation of neutrophil extracellular traps (NETs) through the ERK5 pathway. (A) Heat map image of microarray analysis of RNA from human neutrophils (*n* = 3/group). (B) The mRNA expression levels of representative MAPK family genes in neutrophils. (C) The mRNA expression levels of *MAPK7* in neutrophils in different time limits. (D) Quantification of dsDNA in neutrophils. (E) Representative immunofluorescence images staining for MPO (green), NE (red), and DAPI (blue) of neutrophils, scale bar = 30 μm. (F) The protein expression of MPO in neutrophils, tested by ELISA. (G) Quantification of dsDNA in neutrophils. (H) Phenotypic presentation of back skin sections of sham‐ or IMQ or/and AX‐15836‐treated mice, scale bar = 5 mm. (I) The H&E staining of back skin sections of sham‐ or IMQ or/and AX‐15836‐treated mice, scale bar = 50 μm. (J) The epidermal thickness of mice dorsal skin. (K) Clinical scores of mice dorsal skin

To determine the regulatory link between SHP2 and NETs, neutrophils were extracted from human peripheral blood and treated with IMQ or/and SHP099 or no treatment at all. RNA sequencing showed that: genes encoding proteins involved in the formation of NETs were highly expressed after IMQ treatment, such as *MPO*, *ELANE*, and *PADI4*; Genes encoding inflammatory factors, such as *IL1B* and *IL6* were highly expressed too after IMQ treatment, whereas SHP099 reversed the phenomenon. In addition, genes related to NETs formation were enriched, such as MAPK family, *AKT1*, *AKT2*, *GSDMD*, and so on (Figure 7A). Previous studies have reported that the MAPK pathway is involved in forming NETs.^30^ Therefore, we used qPCR to explore four major MAPK family classifications: ERK, ERK5, JNK, p38 MAPK, which were encoded by *MAPK1*, *MAPK7*, *MAPK8*, and *MAPK14*, respectively. Results showed that *MAPK7* was significantly overexpressed after IMQ treatment (Figure 7B). Then, for 30 min, 60 min, or 120 min, we treated neutrophils with IMQ or/and SHP099 or none, and discovered that SHP099 significantly decreased the elevated *MAPK7* expression generated by IMQ, and the drop was more pronounced with a longer treatment time (Figure 7C).

Next, neutrophils were treated with AX‐15836 (ERK5 inhibitor) and found that the formation of NETs was inhibited, which is similar to the effect of SHP099 (Figure 7D,E). Finally, we treated neutrophils with lovastatin, which has been reported to elevate the SHP2 activity in cells.^31^ The results indicated that lovastatin could induce high levels of NET expression, however when SHP2 or ERK5 were inhibited, NETs were no longer formed in significant amounts. This conclusion was corroborated by ELISA‐detected MPO protein expression. (Figure 7F), as well as dsDNA quantitative detection (Figure 7G). As a result, SHP2 may promote the formation of NETs through the ERK5 pathway.

In addition, AX‐15836 (15 mg/kg) was used on mice to further study the effect of ERK5 in psoriasis. The results showed that psoriasis symptoms caused by IMQ on the back of mice skin were improved in AX‐15836‐treated mice (Figure 7H). As we could see in H&E staining, the symptom of increased epidermal thickness was also relieved after inhibiting ERK5 in mice (Figure 7I,J). Accordingly, the PASI score of the AX‐15836‐treated mice was significantly lower than IMQ‐treated mice (Figure 7K).

It was worth emphasizing that single‐cell RNA sequencing of mice revealed that neutrophils interact with a variety of cells, including fibroblasts, neutrophils, and Schwann cells. Results in Figure S4A displayed a strong interaction between neutrophils and keratinocytes. Then, we used human single‐cell RNA sequencing data and analyzed the interactions between keratinocytes and other cells. This investigation indicated that keratinocytes communicate with immune cells, notably T cells, followed by neutrophils (Figure S4B). Psoriasis has been linked to abnormal keratinocyte proliferation.^32^ We collected keratinocytes from mice skin single‐cell RNA sequencing data (Figure S4C,D) and performed analysis, which revealed that IMQ increased the expression of keratinocyte proliferation‐related genes, such as *Krt77*, but SHP099 decreased their expression (Figure S4E). Further study revealed that IMQ increased the expression of genes involved in keratinocyte proliferation that are abundant in the hedgehog and PI3K pathways, while SHP099 reversed the effect (Figure S4F). We hypothesize that IMQ‐induced NETs release may promote keratinocyte proliferation, which results in psoriasis, and that this effect may be controlled by the hedgehog and PI3K pathways.

## 3 DISCUSSION

Psoriasis's pathophysiology has long been a puzzling conundrum. It has proven difficult to describe how keratinocytes and different immune cells interact to form inflammatory and immunological loops. This loop mediates psoriasis’ initiation and progression. Patients with psoriasis develop skin scales as a result of aberrant keratinocyte growth, which makes keratinocytes the focal point of psoriasis.^33^

In the early stages of psoriasis, keratinocytes are believed to generate an innate immune response.^34^ Psoriasis begins with keratinocytes recruiting neutrophils to penetrate the skin tissue and activate innate immunity. Keratinocytes also recruit dendritic cells, which deliver antigens to T cells, activating adaptive immunity.^34^ LL37 cathelecidin and lipid antigens are determined to be autoantigens derived from keratinocytes, which leads to the activation of T cells, especially the T cells that produce IL‐17 and IL‐23. The release of various inflammatory factors leads to the amplification of local immune response, which results in the confusion of the skin immune system in patients with psoriasis.^33^

Accumulating evidence indicates that SHP2 is associated with autoimmune diseases, such as systemic lupus erythematosus.^35^ Wang et al. found that SHP2 inhibition could relieve systemic lupus erythematosus by reducing the number of double‐negative T cells, regulating ERK/MAPK signal, and reducing IFN‐γ production.^35^ In these situations, SHP2 usually plays a role in aggravating the disease. As a kind of autoimmune disease, psoriasis has caused severe harm to the body and psychology of patients.^36^ Recently, the relationship between SHP2 and psoriasis was also elucidated by our group in terms of macrophage.^28^ We revealed a novel regulation by SHP2 of TLR7‐mediated NF‐κB signaling activation in macrophages. Such positive regulation by SHP2, through dephosphorylation of TLR7 at Tyr1024, promotes the trafficking of TLR7 to the endosome and maintains excessive activation of TLR7/NF‐κB signaling, thus aggravating skin inflammation.^28^, ^37^ In addition, NETs’ formation is increased in both peripheral blood and lesion skin of psoriasis patients and correlates with disease severity.^38^ However, whether there is a regulatory relationship between SHP2 and NETs in neutrophils and how SHP2 influences NETs to promote psoriasis remains unclear. Our results suggest that, in neutrophils, SHP2 acts as a critical factor in the pathogenesis of psoriasis and can promote the generation of NETs and increase the expression of inflammatory cytokines associated with psoriasis through the ERK5 pathway. This study highlights that the ERK5 pathway is a potential therapeutic target for the treatment of psoriasis.

The association of SHP2 with other skin‐related diseases has been also studied. For example, SHP2 in keratinocytes was primarily responsible for the UVB‐mediated dephosphorylation of STAT3 and may protect against UV skin carcinogenesis.^39^ Zhu et al. showed that SHP2 in both human keratinocyte cell line (HaCaT) and human primary epidermal keratinocytes could be stimulated by IL‐22 and promoted the activation of extracellular signal regulated kinase 1/2 (ERK1/2), which may affect the progression of psoriasis.^40^ These studies supported that SHP2 in keratinocytes played an important role and might lead to skin diseases. In addition, SHP2 was involved in the process of skin fibrosis. Distler et al. characterized SHP2 as a TGF*β*’s molecular checkpoint, which blocks JAK2/STAT3 signaling. SHP2 was reported to serve as a potential target for treating skin fibrosis.^41^

It is well known that neutrophils play an important role in inflammation‐related disorders. Neutrophils are the most common leukocytes in the blood and one of the earliest responsible immune cells to fight infection. In a mouse model of LPS‐induced acute lung injury, neutrophils can play a key role in the pathogenesis of acute lung injury by promoting inflammation and injury to the alveolar microenvironment. Zhang et al. found that targeted inhibition of SHP2 in neutrophils could alleviate lung inflammation.^42^ In addition, Muraro et al. revealed the relationship between MAPK family and NETosis: the respiratory syncytial virus induces NETosis through PI3K/Akt, ERK and p38 MAPK signaling,^43^ which leads to respiratory diseases in infants. In the present study, we demonstrated that SHP2 could promote NETosis in neutrophils through the ERK5 pathway, which exacerbates psoriasis progression.

In conclusion, our study combines single‐cell RNA sequencing, spatial transcriptome analysis, RNA‐seq analysis, and experimental verification to provide strong evidence that SHP2 promotes NETosis in neutrophils through the ERK5 pathway. SHP2 inhibition can significantly ameliorate psoriasis‐like skin inflammation in mice, suggesting SHP2 may be a potential therapeutic target for the treatment of psoriasis.

## 4 MATERIALS AND METHODS

### 4.1 Animal experiments

All the experimental animals were purchased from GemPharmatech Co. Ltd., and animals were maintained in specific pathogen‐free conditions at GemPharmatech Co. Ltd and Experimental Animal Center at Nanjing University. *Shp2* ^flox/flox^ mice were crossed with S100a8‐cre mice on the C57BL/6 background for more than two generations to generate *Shp2* ^flox/flox^ S100a8‐cre mice which SHP2 was conditional knocked out in neutrophils. We also constructed *Padi4* knockout on the C57BL/6 background.

Mice were shaved and treated with imiquimod (GTH110C, 3 M Health Care Limited, UK) or petroleum. For different experimental needs, 10 mg/kg SHP099 (HY‐100388, MCE, USA), 15 mg/kg AX‐15836 (T14360, TopScience, China) or physiological saline solution, was injected intraperitoneally (*i.p*.). Serum was isolated from the blood of mice anesthetized with 0.05 mg/kg pelltobarbitalum natricum, and followed by retro‐orbital sinus taken. Skin and serum were stored at −80°C for future use.

### 4.2 PASI score

The mice's dorsal skin changes were observed and calculated according to the PASI scoring standard for the degree of erythema, scaling, and infiltration. The PAIS scores range from 0 to 4 points, 0 points: none; 1 point: mild; 2 Points: moderate; 3 points: obvious; 4 points: extremely obvious.

### 4.3 Neutrophils isolation and cell culture

Ficoll‐Hypaque density gradient centrifugation was used to isolate neutrophils from the peripheral blood of healthy donors. According to the instructions of the Human Neutrophil Isolation Kit (LZS11131, Haoyang Biology, China), neutrophil separation solution of different concentration gradients has been added in advance to the sterile silicified centrifuge tube. Peripheral blood and hydroxyethyl starch were mixed and then superimposed on the separation solution. After centrifugation, neutrophils were pipetted out, followed by washing, centrifugation, and resuspending. Neutrophils were planted in 9 cm cell culture dishes with RPMI 1640 Medium (01‐100‐1A, Biological Industries, Israel), 10% fetal bovine serum (2033119, Biological Industries, Israel), and 1% penicillin‐streptomycin (C0222, Beyotime, China). Cells grew in an incubator at 37°C with 5% CO_2_. Neutrophils are treated with IMQ (10 μM, HY‐B0180, MCE, USA), and/or SHP099 (1, 3, 10 μg/ml, HY‐100388, MCE, USA), and/or AX‐15836 (10 μM, HY‐101846, MCE, USA) or none.

### 4.4 Immunohistochemistry

The immunohistochemistry experiment was performed according to the instructions of the immunohistochemistry detection kit (PK10006, Proteintech Group, USA). Slides were treated with 5% sodium citrate buffer (P0081, Beyotime, China) and 2% hydrogen peroxide for antigen retrieval and endogenous peroxidase activity blocking. Three percent goat serum was used to block non‐specific binding sites of antibodies. Then, the slides were incubated with the primary antibody: rabbit anti‐MPO antibody (ab208670, Abcam, USA), mouse anti‐NE antibody (sc‐55549, Santa Cruz Biotechnology, USA) at 4°C in a humidified chamber overnight. The next day, slides were treated with the second antibody. The hematoxylin was used to observe the nucleus. Finally, the slides were imaged by a light microscope (IX61, Olympus, Japan).

### 4.5 Immunofluorescence

Immunofluorescence staining was performed on tissue and cell samples. For tissue samples, slides were deparaffinized, rehydrated, and treated with sodium citrate buffer for antigen retrieval. Slides were incubated with 3% goat serum for 30 min followed by primary antibodies. Cell samples were fixed in poly‐l‐lysine polylysine (P2100, Solarbio, China), followed by permeabilization and non‐specific binding site blocking. Samples were treated at 4°C overnight with the following primary antibodies: rabbit anti‐MPO antibody, mouse anti‐NE antibody, goat anti‐shp2 antibody (ab9214, Abcam, USA), rabbit anti‐pad4 antibody (ab214810, Abcam, USA), and rabbit anti‐histone H3 (citrulline R2) antibody (ab174992, Abcam, USA). The next day, Samples were incubated with an Alexa fluorescein‐labeled secondary antibody for 2 h. DAPI (C1002, Beyotime, China) was used to stain cell nuclei. Then, samples were imaged by an inverted confocal microscope (LSM880 with airyscan, Carl Zeiss, Germany).

### 4.6 Histopathologic assessment

Slides were deparaffinized, rehydrated, and treated with hematoxylin solution and eosin staining according to the instruction of the Hematoxylin‐eosin staining kit (G1005, Servicebio, China) to evaluate pathological changes in skin tissue. Then, slides were dehydrated and sealed with neutral balsam.

### 4.7 Western blotting assay

Protein samples were prepared by tissue lysate and BCA protein assay. Fifteen microliter protein of each sample was loaded and separated by 10% sodium dodecyl sulfate‐polyacrylamide gel electrophoresis (SDS‐PAGE) and transferred onto polyvinylidene fluoride (PVDF) membranes (IPVH00010, Merck Millipore, Germany). The membranes were washed with 5% nonfat milk to block the non‐specific binding sites and then treated with primary antibodies: rabbit anti‐pad4 antibody, rabbit anti‐histone H3 (citrulline R2) antibody, mouse anti‐β‐actin antibody (M20011, Abmart, China) overnight. The secondary antibody was incubated with the membranes for 2 h. Signals were detected and exposed using LumiGLO^®^ Reagent (#7003, Cell Signaling Technology, USA).

### 4.8 DsDNA quantification

Quant‐it PicoGreen dsDNA Assay kit (P7581, Thermo Fisher Scientific, USA) was used to quantify cfDNA in mice serum. Samples, TE buffer, and Quant‐iT PicoGreen reagent were added into a 96‐well black opaque plate. Fluorescence signals were detected by a microplate fluorescence reader (Safire, Tecan, Switzerland) with an excitation wavelength of 480 nm and an emission wavelength of 520 nm.

### 4.9 Enzyme‐linked immunosorbent assay (ELISA)

The expression of IL‐1*β*, TNF‐*α*, IL‐6, CXCL‐15, NE, and MPO was detected by the DuoSet ELISA kit (R&D System, USA). Briefly, the capture antibodies were coated in a 96‐well enzyme‐linked immunoassay plate (Costar 3590, Corning Incorporated, USA) on the first day at room temperature. After being treated with washing buffer, skin lysate and serum were incubated in the plate for 2 h. Then, the detection antibodies were treated, and absorbance at 450 nm was detected by a microplate (Safire, Tecan, Switzerland).

### 4.10 Quantitative polymerase chain reaction (qPCR)

Trizol was used to extract total RNA (108‐95‐2, Takara, China). After quality control, we quantified 1 μg RNA and reversed transcribed it to synthesize single‐stranded complementary deoxyribonucleic acid (cDNA). We performed quantitative PCR for CFX 100 (Hercules, Bio‐Rad, USA) cyclers using the primers listed in Table S1. The procedure of the amplification program was: 95°C for 2.5 min, 95°C for 15 s, 60°C for 30 s, for a total of 44 cycles. Dissociation curves were analyzed at the end of amplification. RNA expression levels of GAPDH were used for normalization.

### 4.11 RNA‐seq analysis

Neutrophils from peripheral blood of healthy donors were stimulated with IMQ or/and SHP099 or none for 2 h. Total RNA was isolated with Trizol, and subjected to RNA‐seq analysis. RNA integrity was assessed using the RNA Nano 6000 Assay Kit of the Bioanalyzer 2100 system (Agilent Technologies, USA). PCR was performed with Phusion High‐Fidelity DNA polymerase, Universal PCR primers and Index (X) Primer. At last, PCR products were purified (AMPure XP system), and library quality was assessed on the Agilent Bioanalyzer 2100 system. The clustering of the index‐coded samples was performed on a cBot Cluster Generation System using TruSeq PE Cluster Kit v3‐cBot‐HS (Illumia). After cluster generation, the library preparations were sequenced on an Illumina Novaseq platform (Novogene, China).

### 4.12 Single‐cell RNA sequencing

#### 4.12.1 The skin tissue dissociation

We use collagenase I (Sigma, USA), collagenase II (Sigma, USA), Dispase® (Sigma, USA), and mechanical cutting to dissociate the mice skin tissue into single‐cell suspensions. We use the Whole Skin Dissociation Kit (Miltenyi Biotec, Germany) to digest the human skin tissue.

#### 4.12.2 Sequencing library preparation and single‐cell RNA sequencing

10× Genomics Chromium Single Cell 3′ Solution was used to capture single cells. We constructed the RNA‐seq libraries by following the 10× Genomics’ protocol. Then, the resulting libraries were sequenced in a NovaSeq 6000 System (Illumina, USA). The scRNA‐seq data have been uploaded in the GEO database: accession number GSE165021.

#### 4.12.3 Mouse scRNA sequencing processing

The Seurat R package (v4.1.1) was used for filtering cells, data normalization, dimension reduction, and unsupervised clustering. We filtered cells that expressed more than 5,000 genes and less than 500 genes, as well as cells with more than 5% mitochondrial reads. After normalizing each sample, 1,000 features (genes) were selected using the “FindVariableFeatures” function of Seurat. We used “vst” method in Seurat to integrate data to correct for batch effect. The RunPCA function was applied to reduce the dimensionality of datasets. Then, the main cell clusters were identified by the FindClusters function. The uniform manifold approximation and projection (UMAP) were used for visualizing the clusters. We used the ‘‘FindAllMarkers’’ function to detect differentially expressed genes among clusters and chose the top 30 differentially expressed genes (DEGs) for cluster definition.

#### 4.12.4 Human scRNA sequencing processing

The raw scRNA‐seq data were processed with CellRanger (version 6.0.2), and the reads were aligned to hg38 genome. We filtered cells that expressed more than 5,000 genes and less than 100 genes, as well as cells with more than 10% mitochondrial reads. We gained 18,013 cells (normal: 13,681, psoriasis: 4,332). After normalizing each sample, 2,000 features (genes) were selected using the “FindVariableFeatures” function of Seurat. We used “vst" method in Seurat to integrate data in order to correct for the batch effect. We conducted principal component analysis (PCA) for dimensional reduction and chose the top 18 PCs for neighborhood graph computing. The UMAP was used for visualizing the clusters. We used the ‘‘FindAllMarkers’’ function to detect differentially expressed genes among clusters and chose the top 30 DEGs for cluster definition.

#### 4.12.5 Enrichment GO categories

We used the 3,000 genes, which were used for integration for gene set enrichment analysis (GSEA). Using cluster profile (R version: 3.11.1), we performed the analysis based on the scaled expression of genes, with the function: gseKEGG. We show the result by function DimPlot in R package Seurat.

#### 4.12.6 Cell–cell interaction analysis

CellChat was used to analyze intercellular communication networks of each cell type. This tool was used to compute the communication at the signaling pathway level by exploring related ligands/receptors. The contribution of each ligand–receptor pair to overall signaling pathways was computed and visualized by a bubble plot.

#### 4.12.7 Spatial transcriptomics

The spatial transcriptomics’ (ST) sample consisted of two skin sections from control and one lesion skin from a psoriasis patient. We processed the samples using ST experiment (10× Genomics), following the manufacturer's instructions. After cDNA amplification and cDNA library construction, we sequenced the resulting libraries in a NovaSeq 6000 System (Illumina, USA).

#### 4.12.8 Human spatial transcriptomics analysis

The raw spatial transcriptome data were processed with spaceranger (version 1.2.0), and the reads were aligned to the human reference genome (version: GRCh38). The matrix included 2,006 cells from the normal spots and 3,258 cells from the psoriasis spots. SCTransform was conducted to normalize the data and then conducted PCA to integrate the two spots, using 3,000 genes. PCA was performed for dimensionality reduction. We chose the first 30 PCs for clustering and chose UMAP for visualization. Also, we used the ‘‘FindAllMarkers’’ function to detect differentially expressed genes among clusters and chose the top 30 DEGs for cluster definition.

### 4.13 Statistics

Data are represented mean ± SEM. *P* values are determined by two‐tailed unpaired Student's *t*‐test. Differences were considered significant at *P < 0. 05 or **P < 0.01.

## CONFLICT OF INTEREST

The authors declare that there is no conflict of interest that could be perceived as prejudicing the impartiality of the research reported.

## ETHICS APPROVAL

**Mice**: All the procedures were carried out following the Guide for the Care and Use of Laboratory Animals (National Institutes of Health, USA) and ethical regulations of Nanjing University to guarantee animal welfare, approval No: IACUC‐2201003.

**Human specimens**: Psoriasis patients and normal healthy donors' skin and blood sample acquisitions were approved by the Ethics Committee of West China Hospital under protocol 2019‐R‐513. Written informed consent has been obtained from all patients.

## AUTHOR CONTRIBUTIONS

Yang Sun, Jiao Qu and Haiyan Sun conceived the project and designed the study. Yan Ding, Zijun Ouyang and Yuyu Zhu performed experiments. Chenyang Zhang and Jiao Qu analyzed the data. Qiang Xu provided experimental materials and scientific suggestions. Yan Ding, Jiao Qu and Yang Sun wrote the manuscript. All authors reviewed the manuscript and discussed the work.

## Supporting information

Supporting Information

## Data Availability

All data are available from the corresponding authors upon request.
