# Supplementary material for: Tyrosine phosphatase SHP2 exacerbates psoriasis‐like skin inflammation in mice via ERK5‐dependent NETosis
Source: MedComm (2020). 2022 Mar 4;3(1):e120. doi: 10.1002/mco2.120 (PMC8906448; doi:10.1002/mco2.120)
Supplement: Supplementary file 1 — Supporting Information [file MCO2-3-e120-s001.docx]

**Supplementary files**

**T****yrosine phosphatase SHP2 exacerbates psoriasis-like skin inflammation in mice via ERK5-dependent NETosis**

Yan Ding^1,#^, Zijun Ouyang^2,#^, Chenyang Zhang^1,#^, Yuyu Zhu^1^, Qiang Xu^1^, Haiyan Sun^2,^ *, Jiao Qu^1,^*, Yang Sun^1,3,^*

**
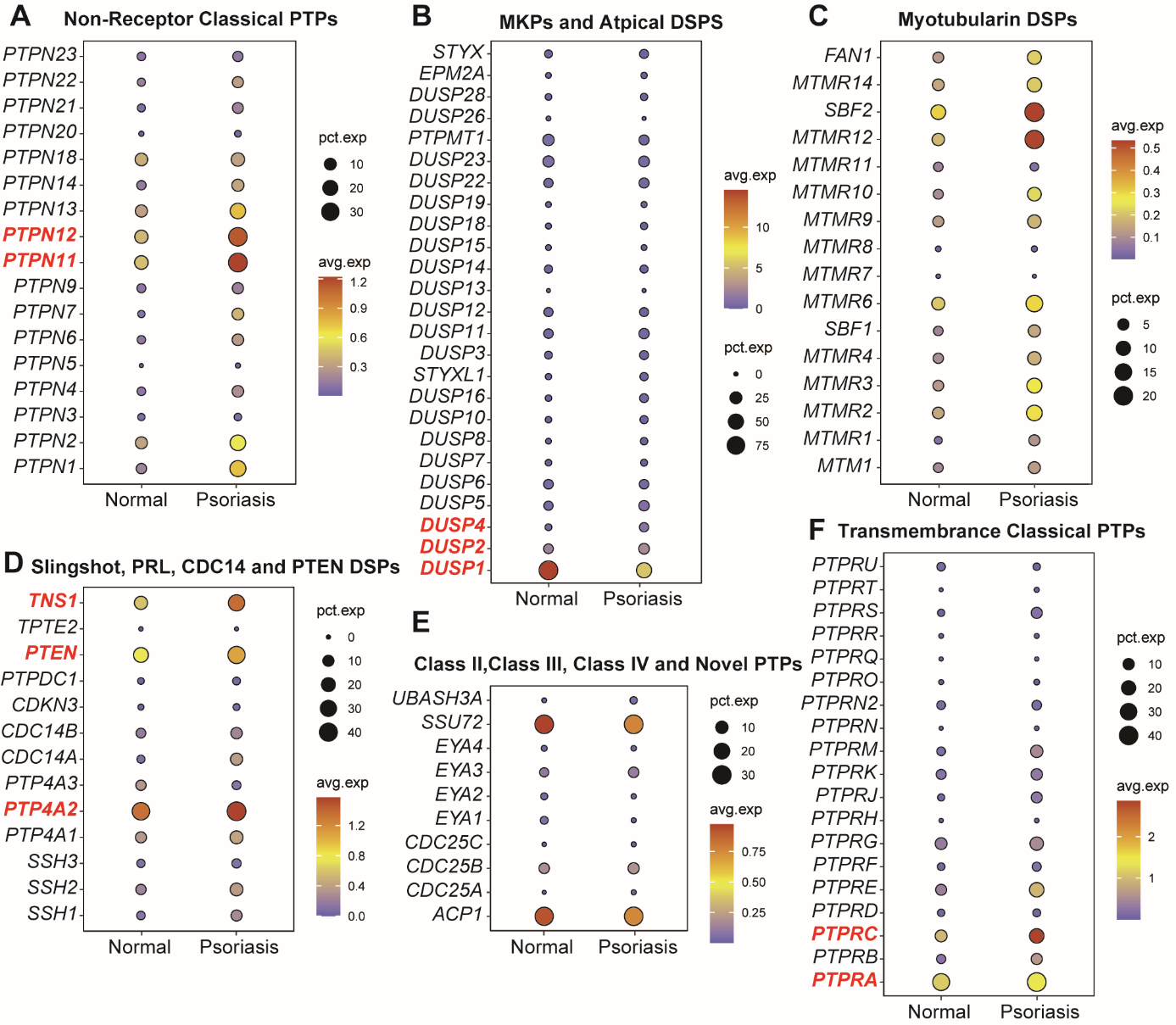
**

**Supplementary Figure 1. Bubble chart of the expression abundance of 107 PTPs which are classified as 6 subtypes**.


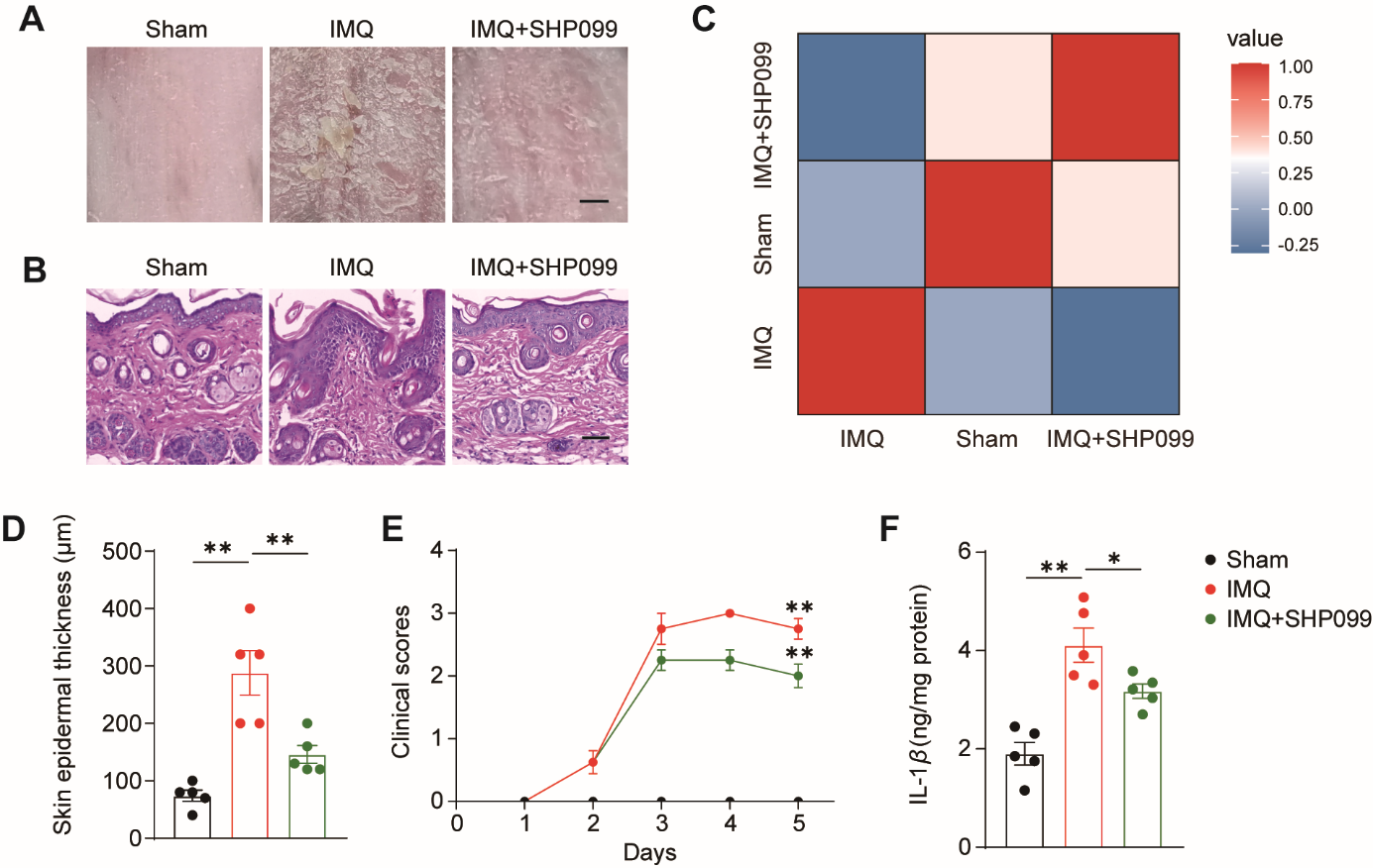


**Supplementary Figure 2. Inhibition of SHP2 can alleviate psoriasis-like symptoms in mice.** (A) Phenotypic presentation of back skin sections of sham-, IMQ- or/and SHP099-treated mice, scale bar=5 mm. (B) The hematoxylin and eosin (H&E) staining of back skin sections of mice, scale bar=200 μm. (C) Pearson correlation between samples. Epidermal thickness (D) or clinical scores (E) of mice dorsal skin. (F) Dorsal skin was infiltered with IL-1*β* evaluated by ELISA.


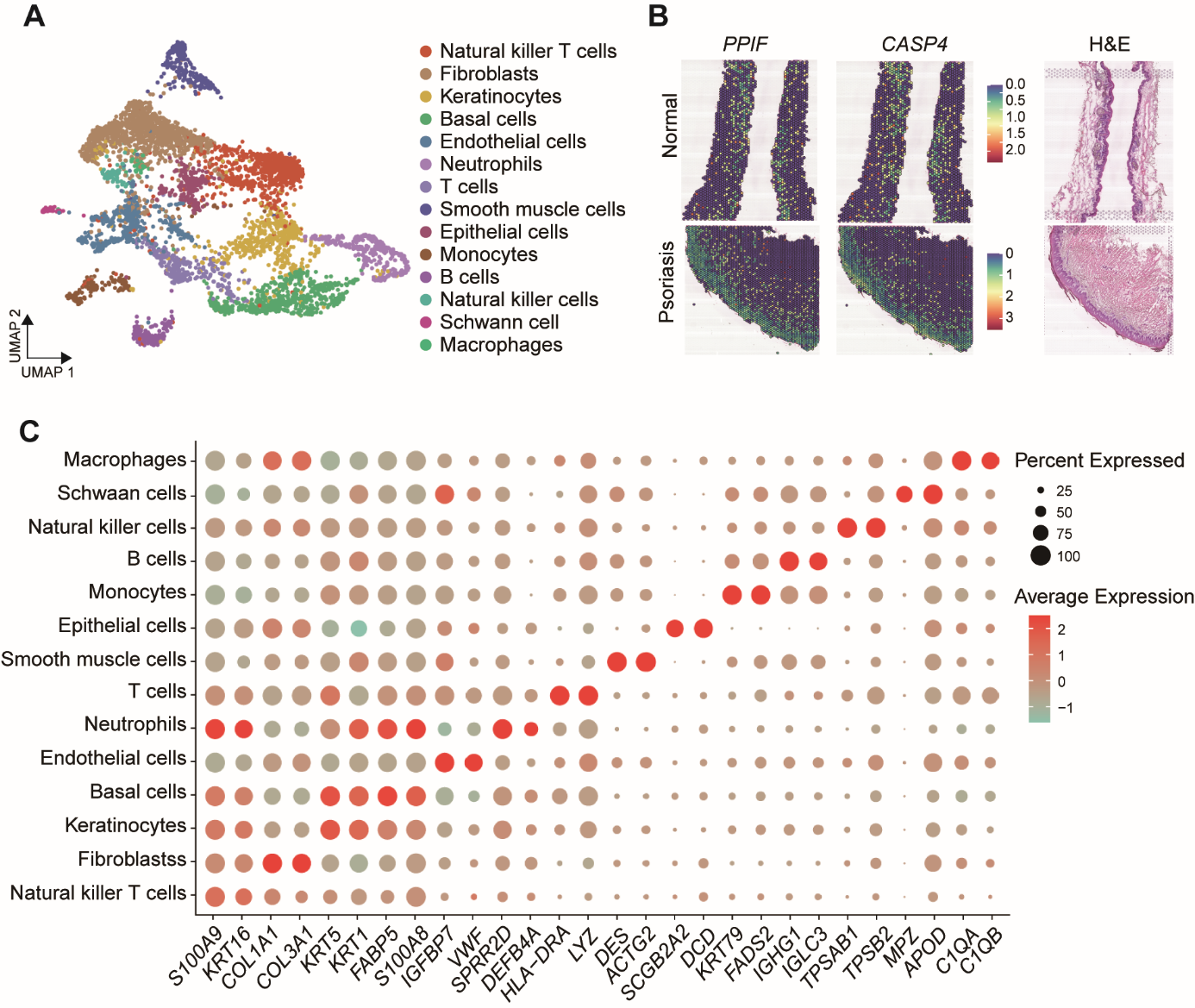


**Supplementary Figure 3. Spatial transcriptomics reveals that NETs exist more in the skin tissue of psoriasis patients.** (A) Unbiased clustering of ST data shown by UMAP plot. (B) H&E staining of the skin sections and spatial feature plot of selected genes’ expression. (C) DotPlot demonstrated the top 2 differentially expressed genes for each cluster.


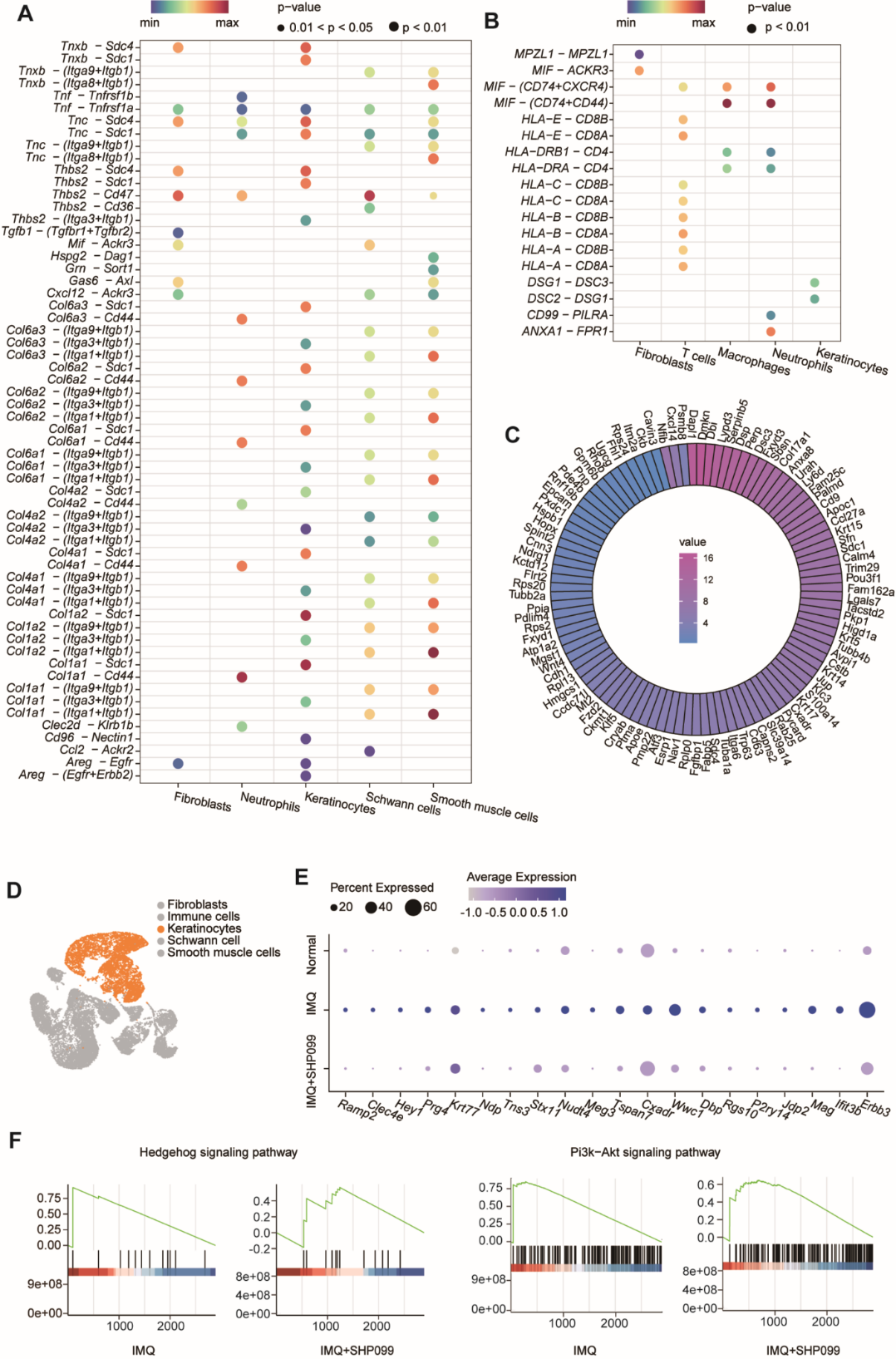


**Supplementary Figure 4. Single-cell RNA sequencing reveals that neutrophils affected keratinocyte proliferation.** (A) Bubble plot of the communication between neutrophils and other cells in mice. (B) Bubble plot of the communication between keratinocytes and other cells in human. (C) UMAP plot displays keratinocytes (yellow) and non- keratinocytes (grey). (D) Ring heatmap showing maker gene in keratinocytes. The top 100 maker genes are showed. (E) Dot plot showing proliferation-related genes in keratinocytes from three groups. (F) Gene set enrichment analysis (GSEA) showing the enrichment of proliferation-related pathways using differentially expressed genes in keratinocytes from three groups.

**Supplementary Table 1. Primers for quantitative PCR analysis**

| Primers | Sequences (5′-3′) |
| --- | --- |
| Human *MAPK1*-F | TACACCAACCTCTCGTACATCG |
| Human *MAPK1*-R | CATGTCTGAAGCGCAGTAAGATT |
| Human *MAPK7*-F | GGTGACTTTGGTATGGCTCGT |
| Human *MAPK7*-R | CCAGAGGTCAATAGCCTGTGTA |
| Human *MAPK8*-F | TGTGTGGAATCAAGCACCTTC |
| Human *MAPK8*-R | TGTGTGGAATCAAGCACCTTC |
| Human *MAPK14*-F | CCCGAGCGTTACCAGAACC |
| Human *MAPK14*-R | TCGCATGAATGATGGACTGAAAT |
| Human *GAPDH*-F | CGTCTTCACCACCATGGAGA |
| Human *GAPDH*-R | CGGCCATCACGCCACAGTTT |
